# Artificial Intelligence in Cardiopulmonary Resuscitation

**DOI:** 10.3390/medicina61122099

**Published:** 2025-11-25

**Authors:** Monica Puticiu, Florica Pop, Mihai Alexandru Butoi, Mihai Banicioiu-Covei, Luciana Teodora Rotaru, Teofil Blaga, Diana Cimpoesu

**Affiliations:** 1 Department of Emergency, Faculty of Medicine, Vasile Goldiș Western University of Arad, 310325 Arad, Romania; puticiu.monica@uvvg.ro (M.P.); pop.florica@uvvg.ro (F.P.); 2 Emergency Medicine and First Aid Department, Faculty of Medicine, University of Medicine and Pharmacy, 200349 Craiova, Romania; mihai.butoi@rmu.smurd.ro; 3 Filantropia Municipal Hospital, 200143 Craiova, Romania; mihai.banicioiu@umfcv.ro; 4 Surgery Department—Emergency Medicine Discipline, University of Medicine and Pharmacy “Grigore T. Popa”, 700115 Iasi, Romania; carmen.cimpoesu@umfiasi.ro; 5 Emergency “St. Spiridon” Hospital, 700111 Iasi, Romania

**Keywords:** artificial intelligence, cardiopulmonary resuscitation, Chain of Survival, emergency medicine

## Abstract

*Background*: Artificial intelligence (AI) and machine learning (ML) have rapidly expanded across the continuum of cardiopulmonary resuscitation (CPR), with growing evidence of their contribution to improving early recognition, intervention quality, and post-cardiac arrest outcomes. This narrative review synthesizes the current advancements and challenges in AI/ML-enhanced resuscitation science. *Methods*: A targeted literature search was conducted in Web of Science for the period 2018–2025 using the keywords “artificial intelligence” and “cardiopulmonary resuscitation”. The search identified studies addressing AI/ML applications across the resuscitation pathway, which were reviewed and categorized according to the American Heart Association’s Chain of Survival—prevention and preparedness, activation of the emergency response system, high-quality CPR including early defibrillation, advanced resuscitation interventions, post-cardiac arrest care, and recovery. *Results*: The literature demonstrates substantial promise for AI/ML in several domains: (1) early recognition and timely activation of emergency medical services through real-time detection algorithms; (2) optimization of high-quality CPR, including feedback systems, automated assessment of chest compressions, and prediction of defibrillation success; (3) support for advanced resuscitation interventions, such as rhythm classification, prognostication, and intra-arrest decision support; (4) post-cardiac arrest care, including outcome prediction and neuroprognostication; and (5) integrative and cross-domain approaches that link multiple phases of resuscitation into end-to-end AI-supported systems. Emerging work also highlights the role of AI in education and training, with applications in simulation, assessment, and skill reinforcement. *Conclusions*: AI/ML technologies hold significant potential to augment clinical performance across all links of the Chain of Survival. Their effective implementation requires attention to ethical considerations, data representativeness, and real-world validation. Future research should prioritize multicenter datasets, transparency, bias mitigation, and clinically embedded evaluation frameworks to ensure that AI/ML systems support safe, equitable, and high-impact resuscitation care.

## 1. Introduction

Cardiac arrest remains a major global public health challenge. Despite successive updates of international resuscitation guidelines and system-level investments, gains in survival—especially when favorable neurological recovery is considered—have been modest [1]. Out-of-hospital cardiac arrest (OHCA) demands immediate recognition, early cardiopulmonary resuscitation (CPR), and timely defibrillation; yet, delays in detection, variability in CPR quality, and limitations in prognostication persist across systems of care [1].

In Europe, the EMS-attended OHCA incidence is reported around ≈50–55 per 100,000 inhabitants, with substantial regional variability [1]. Global comparisons from the International Liaison Committee on Resuscitation (ILCOR) show broad ranges across registries: EMS-treated OHCA 30.0–97.1 per 100,000, with bystander CPR 19.1–79.0%, bystander AED use 2.0–37.4%, and survival to discharge or 30-day survival 3.1–20.4% [2]. A more recent ILCOR follow-up reported 3-year trends and persistent heterogeneity among regions worldwide [3]. For in-hospital cardiac arrest (IHCA), European estimates cluster around 1.5–2.8 per 1000 hospital admissions [4]. These figures underscore both the scale of the problem and the ceiling effects reached by conventional approaches.

The Chain of Survival framework emphasizes early recognition and emergency activation, early CPR, rapid defibrillation, high-quality advanced life support, and integrated post-cardiac arrest care; a sixth link—recovery—was added to highlight long-term survivorship and rehabilitation needs [1,5]. Even within this structure, outcome improvements are constrained by (i) the speed and accuracy of arrest recognition, (ii) real-time optimization of CPR quality and defibrillation decisions, and (iii) reliable neurologic prognostication after return of spontaneous circulation (ROSC) [1].

In this context, AI in resuscitation is evolving from a theoretical innovation to a practical adjunct that can support clinicians and lay rescuers alike. By enabling continuous rhythm monitoring, automated feedback, and adaptive decision-making, AI-driven systems may ultimately enhance the precision and efficiency of life-saving interventions during cardiac arrest.

The application of artificial intelligence (AI), particularly deep learning, in cardiopulmonary resuscitation (CPR) and defibrillation represents a rapidly evolving frontier in emergency medicine. Recent reviews have mapped the landscape of AI in resuscitation, identifying rhythm analysis, outcome prediction, and real-time decision support among the key domains of impact [6]. In the context of out-of-hospital cardiac arrest (OHCA), the timely and accurate classification of shockable versus non-shockable rhythms is critical—yet traditional ECG interpretation, especially during ongoing chest compressions, is hampered by noise and interruptions [7].

To overcome these limitations, multiple recent studies have developed deep neural networks capable of performing rhythm classification even in the presence of compression artifacts. For instance, a multiclass deep learning framework was able to distinguish shockable rhythms, asystole, and organized rhythms from ECGs recorded during mechanical chest compressions, achieving accuracies above 88% despite artifacts introduced by the compression device [8]. In comparative investigations, one-dimensional convolutional neural networks (1D-CNNs) have demonstrated superior performance to recurrent models in classifying cardiac arrest rhythms, achieving accuracies over 90% even on compression-affected ECG segments [9].

Beyond classification, AI offers the potential for “on-device” decision support directly integrated into automated external defibrillators (AEDs). Some earlier efforts have embedded convolutional neural network models in miniature form within AED prototypes to classify shockable arrhythmias in near real time [10].

While those preliminary proofs of concept highlight feasibility, current research continues to address challenges such as robustness to signal noise, real-world generalizability, interpretability, regulatory compliance, and seamless integration into the resuscitation [11].

Integrating artificial intelligence into resuscitation represents a conceptual shift from viewing CPR as a sequence of isolated actions to recognizing it as a continuous decision environment in which information must be processed rapidly, consistently, and with minimal latency. Within this broader perspective, the purpose of this narrative review is to examine how AI technologies have been explored across the major domains of cardiopulmonary resuscitation. Specifically, the review outlines the principal areas in which AI has been applied—early recognition of cardiac arrest, support for bystanders and pre-hospital responders, in-hospital resuscitation processes, and post–resuscitation clinical decision support—without anticipating or reporting specific findings in this introductory section. In parallel, the review identifies the methodological, ethical, and implementation considerations that shape the development and integration of AI systems in resuscitative care.

## 2. Methodology

A narrative review methodology was used to synthesize current evidence on artificial intelligence in cardiopulmonary resuscitation. A targeted search was performed in Web of Science for the period 2018–2025 (cut-off date: 1 October 2025), using the keywords artificial intelligence and cardiopulmonary resuscitation. The search identified 88 articles, of which 56 met the inclusion criteria. Studies were included if they addressed AI applications relevant to any component of cardiac arrest management and were published in English.

A total of 32 studies were excluded for the following reasons: non-English language (n = 2), animal studies (n = 3), conference abstracts without extractable data (n = 7), purely technical AI modeling with no clinical applicability (n = 10), and other causes (n = 10: retracted, 1; education-only, 5; miscellaneous, 4). Selection was performed independently by two reviewers; disagreements were resolved through discussion and, if needed, by adjudication from a senior author.

Although the Chain of Survival can be adapted to specific patient categories, we used the general six-link American Chain of Survival to structure the results. Within the included literature, several special populations were identified: newborns (2 studies), pregnant people (1 study), trauma patients (3 studies), drowning victims (1 study), pediatric patients (1 study).

Because these categories appeared only sporadically across studies, they were integrated into the broader synthesis rather than analyzed in separate sections.

The results are therefore presented according to the six classical links of the Chain of Survival—prevention, early recognition, basic life support, advanced life support, post-resuscitation care, and recovery—to provide a coherent and comprehensive overview of AI applications across the full continuum of cardiac arrest management.

## 3. Chain of Survival

The “Chain of Survival” represents the conceptual and operational backbone of resuscitation science—a sequence of interdependent actions that collectively determine survival after cardiac arrest. Initially introduced by the American Heart Association (AHA) and later adapted by the European Resuscitation Council (ERC), this framework has evolved from four essential links—early recognition, early cardiopulmonary resuscitation (CPR), early defibrillation, and early advanced life support—to encompass post-resuscitation care and, more recently, recovery and rehabilitation as the sixth link [12]. It proposed a global synthesis of the “Chain of Survival”, illustrating how local adaptations—influenced by geography, demography, and healthcare infrastructure—can affect survival outcomes. This scoping review emphasized that future progress depends not only on clinical excellence but also on system connectivity, equitable access to early interventions, and integration of artificial intelligence (AI) and digital health tools across all links of the chain.

In Europe, the ERC 2021–2025 guidelines now explicitly highlight the use of technology—including dispatcher AI support, smartphone responder networks, drone-delivered defibrillators, and data-driven registries—as mechanisms to strengthen each link. Meanwhile, the AHA’s 2020 and 2025 updates have increasingly focused on community resilience, diversity, and inclusivity, ensuring that every individual, regardless of geography or socioeconomic status, can benefit from a functioning Chain of Survival.

**Figure 1:**
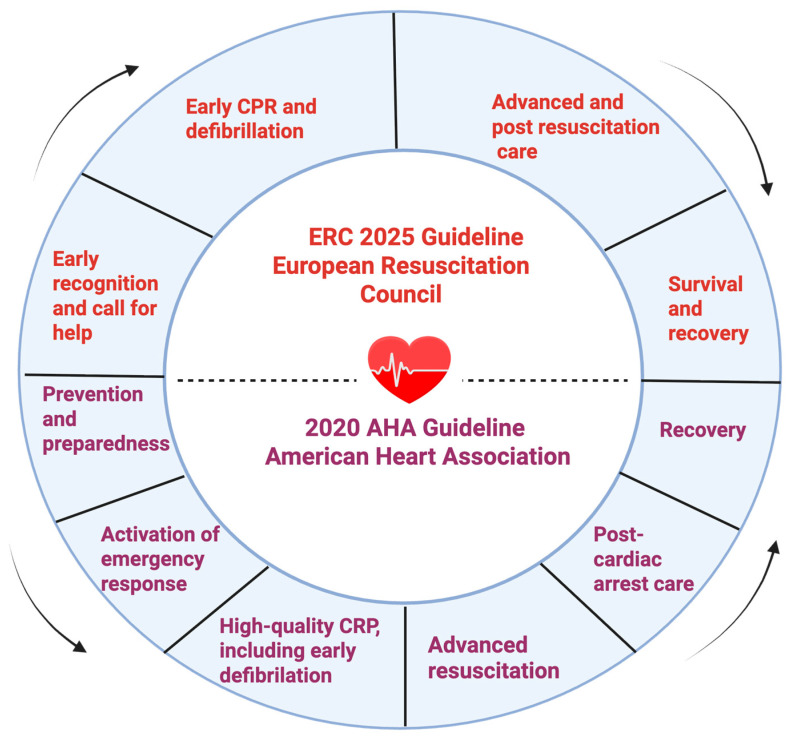
Comparison between the European Resuscitation Council (ERC) and American Heart Association (AHA) Chains of Survival. Created with Biorender.com.

To facilitate comparison, Figure 1 presents the key components of the Chain of Survival as defined by the ERC and AHA, outlining their respective approaches to early recognition, intervention, and post-resuscitation care.

The integration of AI-driven prediction, communication, and feedback tools across its six links marks a paradigm shift—from reactive resuscitation to anticipatory, intelligent, and system-level response to cardiac arrest.

To ensure terminological clarity and consistency across the synthesis, the results are organized according to the American Heart Association (AHA) Chain of Survival, which comprises six sequential components. This structure provides a coherent and comprehensive framework for integrating findings across the diverse body of literature identified. The six components used throughout Section 3 are as follows:

- Prevention and preparedness;
- Activation of the emergency response system;
- High-quality CPR, including early defibrillation;
- Advanced resuscitation interventions;
- Post-cardiac arrest care;
- Recovery.

Although the European and American chains share substantial conceptual overlap, the AHA six-link structure offers a clearer operational categorization that aligns well with the scope and variability of the included studies. Its adoption facilitates a more rigorous and standardized presentation of evidence across the continuum of cardiac arrest management.

### 3.1. Prevention and Preparedness

Prevention and preparedness constitute the first and most foundational link in the Chain of Survival, aiming to identify individuals and populations at risk of cardiac arrest, strengthen system readiness, and ensure that the conditions for an effective response are in place even before collapse occurs. The integration of artificial intelligence (AI) into this domain has redefined prevention strategies—from static and retrospective models toward dynamic, data-driven systems that anticipate risk, allocate resources, and activate tailored preventive actions.

Sudden cardiac death (SCD) remains a major global public health challenge, accounting for a substantial share of cardiovascular and all-cause mortality [13]. Despite advances in cardiac therapies, survival following sudden cardiac arrest (SCA) remains poor, primarily due to the limited predictive accuracy of conventional risk stratification tools such as left ventricular ejection fraction (LVEF). Srivats et al. [13] emphasized the potential of artificial intelligence (AI) and machine learning (ML) to integrate clinical, electrical, imaging, genetic, and biochemical data into multidimensional predictive frameworks, enabling earlier identification of high-risk patients.

AI-based risk models employing deep learning and ensemble algorithms have demonstrated improved sensitivity and specificity for SCD prediction in both in-hospital and community settings, although external validation and population harmonization remain necessary. The review also stressed the importance of system-level preparedness, including automated EMS activation, community responder networks, CPR training, and AED accessibility—all essential to complement predictive technologies.

In obstetric care, unsupervised ML has provided novel preventive insights. A large cohort study of twin pregnancies (n = 823) identified five distinct growth discordance trajectories using k-means clustering, revealing that the “high-stable” pattern correlated with nearly 50% morbidity and 9% mortality [14]. Integrating discordance trajectories with cerebroplacental ratio improved predictive accuracy (AUC = 0.802), demonstrating the potential of ML in early risk stratification and intervention.

Comparable advances are evident in perioperative preparedness. The interpretable Predictive OpTimal Trees in Emergency Surgery (PoTR-ICU) algorithm—derived from over 460,000 surgical cases—accurately predicted postoperative ICU requirements (C = 0.88–0.89) and serves as a real-time triage and decision-support tool to optimize critical care allocation [15].

The capacity of artificial intelligence (AI) to detect precursors of physiological deterioration and trigger preventive action extends into trauma and emergency medicine. The Trauma Outcomes Predictor, based on optimal classification tree algorithms, demonstrated high discriminative accuracy (C = 0.83–0.92) for in-hospital mortality prediction in over 260,000 elderly trauma patients, while also identifying individuals at risk for acute respiratory distress syndrome or cardiac arrest requiring resuscitation [16]. These interpretable machine learning (ML) models exemplify how complex clinical data can be transformed into actionable bedside readiness, enabling clinicians to anticipate deterioration and activate response pathways before critical events occur.

In the prehospital setting, AI-driven telemetry and wearable systems are redefining early ischemia recognition and accelerating emergency activation. A hybrid convolutional neural network–long short-term memory (CNN–LSTM) model deployed in Central Taiwan analyzed real-time prehospital 12-lead ECGs to detect ST-elevation myocardial infarction (STEMI) with exceptional accuracy (AUC = 0.997) [17]. The implementation reduced diagnostic feedback time from 113 s to 37 s and shortened the contact-to-door interval for primary percutaneous coronary intervention to under 20 min. This integration of AI into ambulance-based diagnostics effectively bridges prehospital recognition with definitive therapy, minimizing the delay between ischemic onset and reperfusion treatment.

Preventive strategies are increasingly extending beyond hospital environments, integrating community-based systems and real-time digital infrastructures to enable earlier recognition and faster response. The PROTECTOR system, developed in Poland, exemplifies this paradigm shift toward connected, AI-driven early intervention networks [18]. Using a fuzzy logic–based rhythm classification algorithm, the system detects lethal arrhythmias with 100% sensitivity and 97.8% specificity, instantly triggering audible alarms for bystanders and transmitting geolocation data to emergency medical services (EMS) together with the location of the nearest automated external defibrillator (AED). Its edge-AI diagnostic processor delivers rhythm interpretation within seconds, illustrating how embedded intelligence can seamlessly integrate detection, alarm, and activation. This architecture demonstrates the real-time convergence of prevention and preparedness, offering a scalable model for community-level cardiac emergency readiness.

Parallel advances have emerged within emergency departments, where integrated clinical–AI platforms are enhancing early risk prediction for cardiac arrest. The Medication-informed Cardiac Arrest Early Warning System (MCAEWS) combines pharmacologic profiles with vital signs to identify patients at imminent risk of arrest. Leveraging large emergency department datasets, a random forest algorithm achieved outstanding predictive performance (AUC = 0.98), outperforming both logistic regression and decision tree models [19]. By incorporating medication-related parameters, MCAEWS improved sensitivity and reduced false-positive alerts, thereby reinforcing clinical preparedness and enabling proactive intervention before irreversible deterioration occurs.

Prevention also involves sustained vigilance in highly vulnerable populations. In pediatric cardiac intensive care, where conventional monitoring often fails to identify early decompensation, AI-based predictive analytics can anticipate deterioration and alert teams before catastrophic events [20]. Such models capture complex physiological interactions that precede cardiac arrest, extending preventive capacity beyond human perception and transforming critical care into a continuously adaptive environment.

Preparedness at the population level also relies on predictive tools for systemic complications that amplify mortality risk. A systematic review of models for acute traumatic coagulopathy (ATC) identified four major prediction systems, among which a Bayesian network achieved 90% sensitivity and 92% specificity for identifying patients with abnormal coagulation (PT > 1.2) [21]. Despite varying external validation, these results suggest that AI-driven, interpretable models can meaningfully support early identification of high-risk phenotypes in trauma—a preventive step integral to improving survival after injury and shock.

Table 1 summarizes key studies exploring the role of artificial intelligence (AI) in prevention and preparedness along the cardiac arrest survival chain, highlighting how predictive modeling and automated decision support contribute to early detection and system readiness.

Several of the predictive models identified in the literature contribute directly to cardiac arrest prevention by enabling earlier recognition of physiological deterioration, detecting patterns associated with impending arrest, and alerting clinicians before irreversible collapse occurs. These systems extend the traditional concept of preparedness by shifting part of the response upstream—toward risk stratification, monitoring, and early warning—thereby strengthening the first link of the Chain of Survival. In this way, AI-driven predictive tools support the overarching goal of reducing the incidence of preventable arrests and enhancing readiness across both prehospital and in-hospital environments.

### 3.2. Early Recognition and Activation of the Emergency Medical System

Timely recognition of cardiac arrest and rapid activation of emergency medical services (EMS) represent the critical second link in the Chain of Survival. Artificial intelligence (AI) and digital technologies are reshaping this link by reducing time-to-recognition, improving dispatch accuracy, and facilitating faster coordination between callers, dispatchers, and first responders.

Recent analyses emphasize that technology now permeates every phase of the Chain of Survival—from prediction and prevention to recognition, cardiopulmonary resuscitation (CPR), and defibrillation [26]. Mobile applications alerting nearby responders, drones delivering automated external defibrillators (AEDs), and AI-assisted dispatcher systems have been implemented globally, improving bystander response and survival. Emerging tools such as wearables, smart speakers, and AI-enhanced video or audio surveillance are also being explored for automatic detection of cardiac arrest in both out-of-hospital and clinical environments [26].

Traditional dispatcher-based recognition remains suboptimal, with up to 25% of out-of-hospital cardiac arrests (OHCA) unrecognized during initial emergency calls, leading to missed opportunities for dispatcher-assisted CPR. Machine learning (ML) systems have shown clear potential to augment this process. In a large retrospective analysis of 108,607 emergency calls from Copenhagen, an ML framework outperformed human dispatchers with higher sensitivity (84.1% vs. 72.5%) and faster recognition (median 44 s vs. 54 s) [27]. Similarly, an observational study of 851 OHCA calls reported that ML identified 36% of arrests within the first minute compared with 25% by dispatchers, improving recognition time by 28 s on average (*p* < 0.001) [27]. A randomized clinical trial of 169,049 emergency calls further validated real-time ML support, showing that ML-assisted dispatchers recognized 93.1% of confirmed OHCAs versus 90.5% under standard protocols, while the ML model alone achieved superior sensitivity (85.0% vs. 77.5%) and faster detection [28]. Collectively, these findings confirm AI’s potential as a decision-support layer that enhances early cardiac arrest recognition and accelerates activation of the Chain of Survival.

Beyond acoustic signal recognition, artificial intelligence (AI) is increasingly employed to evaluate emotional and behavioral dynamics during emergency calls. In a study analyzing 337 out-of-hospital cardiac arrest (OHCA) dispatch recordings, an AI model using Mel-frequency cepstral coefficients and support vector machines successfully classified callers’ emotional stability and cooperation levels [29]. The system achieved 98.6% specificity and over 90% predictive accuracy, even when limited to the first 10 s of speech. Such emotion-aware models could pre-screen emotionally stable callers, allowing dispatchers to allocate more attention to distressed individuals where communication barriers may delay cardiopulmonary resuscitation (CPR). Emotion-sensitive AI thus emerges as a promising tool to enhance situational awareness, optimize dispatcher–caller interaction, and improve bystander compliance during resuscitation efforts [29].

While AI integration in emergency medicine enhances diagnostic precision and response efficiency, it also raises significant ethical, social, and regulatory considerations. The European Commission’s High-Level Expert Group on AI has issued guidelines for developing “trustworthy AI”, emphasizing transparency, human oversight, and accountability [30]. A practical application of this framework was demonstrated by an interdisciplinary 1Z-Inspection^®^ assessment—conducted by ethicists, clinicians, policymakers, and legal experts—evaluated an ML-based cardiac arrest detection system deployed in real emergency calls [30].

In Table 2 illustrate the expanding role of AI in supporting rapid activation of the emergency medical system, from automated call analysis and emotional state detection to ethically governed implementation frameworks that ensure trustworthy and accountable clinical decision support.

### 3.3. Basic Life Support (BLS) with High-Quality Chest Compressions and Early Defibrillation

To bridge the gap between human factors and technological solutions, it is important to recognize that many of the challenges observed during BLS—such as variability in CPR quality, delayed recognition, and high cognitive load—are precisely the areas in which AI-based systems have been developed to provide real-time guidance and performance support.

High-quality basic life support (BLS), including effective chest compressions and early defibrillation, remains the cornerstone of survival in out-of-hospital cardiac arrest (OHCA). However, despite continuous educational efforts and technological advancements, bystander CPR rates remain suboptimal worldwide. Understanding and addressing the barriers that prevent laypersons from initiating CPR are essential to strengthening this critical link in the Chain of Survival.

A qualitative investigation by Zhong et al. [32] explored the psychosocial and behavioral determinants influencing the intention and actual performance of CPR among lay rescuers, using the Theory of Planned Behavior (TPB) framework. Through semi-structured interviews with 29 participants, the study identified 11 thematic categories affecting CPR implementation. Emotional resistance to confronting death, fear of legal consequences, and concerns about disease transmission were found to diminish confidence and willingness to intervene. Conversely, recognition of the intrinsic value of saving a life, peer encouragement, and previous training experience increased readiness to perform CPR. Importantly, situational facilitators—such as the presence of AEDs or supportive public environments—were shown to improve real-time decision-making, while lack of self-efficacy and contextual obstacles (e.g., crowded spaces) served as deterrents. These findings suggest that public health strategies must combine psychological preparedness, accessible training, and environmental support to promote sustained community engagement in CPR.

The integration of AI-driven and digital support systems into BLS training and dispatch guidance further enhances lay rescuer performance. The 2022 resuscitation guidelines emphasize technological solutions that strengthen system-level coordination—specifically, telephone-assisted CPR, smartphone-based first responder alerts, and AI-supported video dispatch systems [33]. Telephone CPR (T-CPR) programs now incorporate automated voice recognition and real-time AI coaching to guide compression rate and depth, while mobile applications reduce the “no-flow interval” by notifying nearby trained responders and directing them to AED locations. However, despite proven efficacy, implementation remains inconsistent, and significant regional disparities persist. Moreover, cardiac arrest centers—defined as specialized facilities for post-resuscitation care—require standardized certification processes to ensure quality and equity of care across systems.

Emerging research also demonstrates that AI-enhanced BLS feedback devices and smart defibrillators can improve CPR quality in real time. Automated external defibrillators (AEDs) and implantable cardioverter defibrillators (ICDs) treat life-threatening arrhythmias by classifying electrocardiographic rhythms as shockable or non-shockable [34]. Machine learning (ML) algorithms have improved this process, enabling rhythm analysis without interrupting CPR and enhancing prediction of defibrillation success. Integrating ML into AEDs and ICDs could optimize shock timing, reduce inappropriate shocks, and improve patient outcomes. The creation of large, standardized ECG databases will be essential for validating and safely implementing these technologies in clinical practice.

Collectively, these findings highlight that improving BLS efficacy requires an integrated, multidimensional approach—combining human behavioral insights with AI-driven feedback and coordinated response systems. The convergence of education, behavioral modeling, and intelligent technology not only enhances CPR quality and defibrillation timing but also redefines the role of lay rescuers as active, empowered participants in the continuum of emergency care.

### 3.4. Advanced Life Support (ALS)

Advanced Life Support (ALS) represents the most complex phase of the resuscitation continuum, encompassing real-time cardiac rhythm analysis, defibrillation, airway management, and circulatory support. In this phase, artificial intelligence (AI) and related technologies are reshaping both diagnostic accuracy and therapeutic precision by integrating multimodal physiological data, enabling continuous feedback, and supporting clinical decision-making under time-critical conditions.

A central component of this transformation is the use of prehospital ultrasound (PHUS), which is an emerging diagnostic and therapeutic tool with growing relevance in emergency medicine [35]. When used by trained practitioners, PHUS can support rapid assessment of key prehospital conditions such as respiratory distress, cardiac arrest, shock, and trauma, guiding timely treatment and triage to appropriate specialty centers. Integrated into structured diagnostic algorithms, it enables early identification of life-threatening pathologies and the transmission of real-time images via telemedicine. However, its implementation must avoid delaying critical interventions. The rapid evolution of PHUS technology underscores the need for standardized education and training frameworks to ensure operator competence and skill retention. Despite promising results, current evidence remains limited, with most data derived from observational studies and case reports.

Mechanical chest compression devices have long supported ALS teams in maintaining consistent perfusion during prolonged resuscitation. However, the artifacts generated during compressions often distort electrocardiogram (ECG) readings, complicating rhythm interpretation. Load-distributing band (LDB) mechanical chest compression devices are used during out-of-hospital cardiac arrest (OHCA) but can generate electrocardiogram (ECG) artifacts that interfere with rhythm interpretation. To address this, a deep learning (DL)-based framework was developed for automatic multiclass cardiac rhythm classification—distinguishing shockable, asystole, and organized rhythms—even in the presence of compression artifacts. Using 15,479 ECG segments from 2058 patients, convolutional neural networks (CNNs) and residual networks (ResNets) were compared to traditional machine learning classifiers. The ResNet model achieved the highest performance, with 88.3% sensitivity, 88.3% F1 score, and 88.2% accuracy [8].

Similarly, Ahn et al. [36] developed an artificial intelligence (AI) model to predict shockable cardiac rhythms from electrocardiograms (ECGs) containing chest compression artifacts, using real-world data from emergency department settings. Based on convolutional neural networks and enhanced with gradient-weighted class activation mapping for interpretability, the model analyzed 1889 ECGs from 172 patients who experienced cardiac arrest. It achieved an area under the receiver operating characteristic curve (AUROC) of 0.867, demonstrating robust predictive performance across both manual and mechanical compressions. This represents the first validated AI model capable of identifying shockable rhythms during ongoing CPR, eliminating the need for pauses in compressions. By enabling real-time, explainable rhythm assessment, the model has the potential to reduce treatment delays and improve resuscitation outcomes.

Beyond rhythm detection, ultrasound-based AI systems are enhancing physiologic monitoring and prognostication during resuscitation. Park et al. [37] introduced RealCAC-Net, a deep learning model for quantifying carotid artery compressibility (CAC) to determine return of spontaneous circulation (ROSC). Using over 27,000 ultrasound images, RealCAC-Net achieved a classification accuracy of 96% and an F1 score of 0.97, enabling precise differentiation between effective and ineffective compressions. The authors proposed that real-time integration of CAC-based feedback into resuscitation algorithms could revolutionize ALS by providing objective, image-based confirmation of perfusion during CPR.

At the frontier of circulatory support, extracorporeal cardiopulmonary resuscitation (ECPR) has emerged as a life-saving intervention for refractory cardiac arrest. Tanaka et al. [38] reviewed age-dependent outcomes in ECPR and emphasized the potential of AI to develop predictive models for patient selection and prognosis. The integration of physiological, demographic, and procedural data within machine learning frameworks could optimize decisions about ECPR initiation, balancing survival benefit with ethical considerations in older or comorbid patients. The authors argued that the future of ECPR lies in combining AI-based risk stratification with ethical oversight to ensure equitable access and resource allocation.

Finally, resuscitation research itself is being transformed by the digitalization of defibrillator data. Defibrillator-derived physiological data—such as ECG, thoracic impedance, and end-tidal CO_2_—offer valuable insights into patient response during CPR [39]. However, the absence of standardized data formats and reliance on proprietary systems limit their research potential. The integration of AI-based algorithms for automated rhythm classification and time-dependent modeling can enhance understanding of CPR dynamics and patient outcomes. Harnessing these datasets could inform personalized resuscitation strategies and improve future guideline development, provided that international collaboration establishes open data standards and interoperability across devices.

Collectively, these advancements illustrate that the integration of AI into ALS is not limited to monitoring or diagnosis but extends to real-time interpretation, decision support, and prognostication. By merging human expertise with intelligent automation, AI-enabled systems promise to refine rhythm analysis, optimize defibrillation timing, personalize ECPR indications, and improve outcomes in even the most complex cardiac arrest scenarios. However, as technology assumes a growing role at the resuscitation bedside, sustained focus on training, transparency, and ethical governance remains essential to ensure that AI complements, rather than replaces, clinical judgment.

### 3.5. Post-Resuscitation Care

Post-resuscitation care represents a pivotal phase in the Chain of Survival, determining long-term neurological and functional outcomes after return of spontaneous circulation (ROSC). Despite significant advancements in cardiopulmonary resuscitation (CPR) and early defibrillation, mortality and morbidity after cardiac arrest remain high, primarily due to hypoxic–ischemic brain injury and multisystem dysfunction. Artificial intelligence (AI) and predictive analytics are now playing an increasingly central role in refining prognostication, guiding treatment intensity, and informing ethical and clinical decision-making during post-resuscitation management.

A retrospective study from Northern Jordan examined the incidence and survival outcomes of pediatric in-hospital cardiac arrest (IHCA) over an eight-year period. Among 504 resuscitated children, the incidence was 6.26 per 1000 admissions, with an overall ROSC rate of 25% and survival to discharge of only 4.8% [40]. Notably, survival improved by an average of 24% annually, particularly among patients with bradycardic events and those treated outside intensive care settings [40]. The study emphasized the urgent need for structured post-resuscitation systems, including national registries, pediatric-specific resuscitation pathways, and ethical frameworks for end-of-life decisions. These findings underscore that survival alone is insufficient; the quality of post-resuscitation neurological recovery must become a central outcome metric.

In adult populations, artificial intelligence has begun to reshape prognostic modeling following out-of-hospital cardiac arrest (OHCA). A large-scale study using data from the Swedish Cardiopulmonary Resuscitation Registry applied an interpretable machine learning (ML) approach—the XGBoost algorithm—on over 55,000 OHCA cases to predict neurological outcomes at hospital admission [41]. Using 10 key features (including ROSC at arrival, initial rhythm, and level of consciousness), the model achieved AUC = 0.964, specificity 97.5%, and macro F1 = 0.803, with excellent calibration and explainability via SHapley Additive exPlanations (SHAP) [41]. The integration of explainable AI enables clinicians to visualize the influence of each variable, promoting transparency in decision-making for targeted neuroprotective interventions and resource allocation in the ICU.

Similarly, Kawai et al. [42] developed a deep learning model using post-resuscitation brain CT scans obtained within 3 h after cardiac arrest to predict poor neurological outcomes at one month. Employing transfer learning and heatmap visualization, the model focused on the gray-to-white matter boundary, the key anatomic region for detecting hypoxic–ischemic injury. Compared to traditional gray-to-white matter ratio (GWR) methods, the ML-based approach achieved a higher precision-recall AUC (0.73 vs. 0.58), demonstrating superior predictive accuracy and clinical interpretability. This framework exemplifies how imaging-based AI can complement clinical and electrophysiologic parameters to enhance prognostication after ROSC.

Taken together, these studies highlight a paradigm shift from survival-based metrics to neurologically informed, data-driven post-resuscitation care. Predictive AI models—whether using structured clinical data, physiological monitoring, or neuroimaging—offer an unprecedented ability to stratify risk, personalize therapeutic strategies (e.g., targeted temperature management, hemodynamic optimization), and improve communication with families regarding prognosis.

Beyond their predictive capabilities, such models can also inform ethical frameworks by providing objective, reproducible criteria for withdrawal of life-sustaining therapy, ensuring decisions align with both patient dignity and clinical evidence. To achieve these aims, the development of standardized post-resuscitation registries, international data-sharing protocols, and multidisciplinary collaboration between clinicians, data scientists, and ethicists is crucial.

Ultimately, the integration of AI into post-resuscitation care marks a transition toward precision resuscitation medicine, where early physiological stabilization and long-term neurological recovery are guided by intelligent, interpretable, and ethically governed technologies.

### 3.6. Recovery

The final link in the Chain of Survival, recovery, extends beyond hospital discharge and focuses on optimizing long-term outcomes—neurological, cardiovascular, and psychosocial—for cardiac arrest survivors. The post-cardiac arrest period marks a transition from stabilization to rehabilitation, demanding sustained multidisciplinary care that includes neurological monitoring, physical reconditioning, and psychosocial support. Recent advancements in artificial intelligence (AI) and predictive modeling are transforming this stage, allowing for dynamic outcome prediction, individualized rehabilitation planning, and better allocation of advanced therapeutic resources.

A key area of innovation is in cardiac recovery and transplantation, where resuscitated or marginal donor hearts are increasingly utilized through advanced preservation techniques. Leon et al. [43] highlighted significant progress in donor heart recovery and evaluation technologies, including normothermic machine perfusion, thoraco-abdominal normothermic regional perfusion, and hypothermic oxygenated perfusion. These methods sustain metabolic activity, minimize ischemic injury, and expand the pool of viable donor hearts—including those recovered after circulatory death or post-cardiopulmonary resuscitation. AI-driven assessment tools further refine donor selection and predict graft performance, representing a critical evolution in post-resuscitation recovery that bridges emergency care with long-term cardiac replacement strategies.

AI has also begun to impact forensic and diagnostic evaluation in post-mortem and post-resuscitation contexts. Ogawara et al. [44] developed a deep learning model using postmortem CT scans to distinguish drowning from non-drowning deaths. The modified AlexNet architecture achieved an AUC of 0.95, demonstrating high diagnostic accuracy. Interestingly, model accuracy decreased to 81% in cases where aggressive resuscitation had been performed—highlighting how cardiopulmonary interventions alter pulmonary imaging patterns. This study underscores the importance of contextualizing AI outputs within clinical and procedural histories, a principle equally relevant to rehabilitation imaging and functional recovery monitoring.

In the clinical realm, real-time predictive algorithms are redefining the monitoring of post-resuscitation trajectories. Shin et al. [45] developed the Cardiac Arrest Prediction using Deep learning (CAPD) model, trained on over 16,000 OHCA cases. Incorporating variables such as age, bystander CPR, epinephrine use, and response time, the CAPD achieved AUCs of 0.828 for prehospital ROSC and 0.907 for favorable neurological outcome, outperforming conventional models. Notably, the system demonstrated how increased on-scene time interval (STI) proportionally reduced the likelihood of ROSC and neurological recovery, offering actionable insights for personalized resuscitation strategies and post-event rehabilitation timelines.

Expanding this concept, Kim et al. [46] introduced the Time-Adaptive Conditional Prediction Model (TACOM), a framework capable of minute-by-minute prognosis for patients undergoing in-hospital or emergency department CPR. Based on a dataset of nearly 50,000 patients, TACOM utilized LightGBM as the most accurate algorithm, achieving AUROC values ranging from 0.910 to 0.869 for neurological outcomes and 0.800 to 0.734 for survival. This dynamic, time-sensitive approach reflects a new paradigm in recovery prediction—one where continuous learning systems can adapt to evolving physiological data and guide resuscitation efforts toward optimal long-term recovery potential.

The rapid evolution of AI-enhanced prognostic models integrate early clinical variables—such as initial rhythm, ROSC time, and in-hospital parameters—to predict neurological outcomes after cardiac arrest [47]. While not designed for individual patient decisions, these data-driven scoring systems provide valuable tools for cohort stratification, research design, and rehabilitation planning [47]. Their integration with big data analytics and machine learning holds promise for personalized post-resuscitation care, enabling tailored rehabilitation strategies and optimized resource allocation based on predicted recovery profiles.

Collectively, these studies illustrate a comprehensive shift toward AI-enabled precision recovery after cardiac arrest. From molecular and imaging biomarkers to dynamic predictive systems, artificial intelligence enables clinicians to anticipate recovery patterns, tailor follow-up care, and monitor long-term neurological integrity.

## 4. Integrative and Cross-Domain Approaches in Resuscitation Science

In structuring this review, we initially attempted to group all identified studies exclusively according to the six links of the Chain of Survival. However, a considerable proportion of the literature simultaneously addressed multiple phases of the resuscitation process. Given that resuscitation represents an inherently integrated continuum—whose success depends on the coordinated interaction of prevention, recognition, basic and advanced life support, and post-cardiac arrest care—many AI applications naturally operate across several domains rather than within a single isolated step.

To accurately reflect this multidimensionality, we included a dedicated section on integrative and cross-domain approaches. This section encompasses studies that span two or more components of the Chain of Survival, propose end-to-end AI solutions across the continuum of care, or examine workflow-level interactions that bridge distinct elements of resuscitation practice.

While most contemporary studies on cardiac arrest align with specific links in the Chain of Survival, a growing body of literature transcends these traditional divisions. These multidisciplinary contributions—encompassing telemedicine, artificial intelligence (AI), misinformation, education, and digital transformation—provide a systemic view of how technology and society interact to shape the future of resuscitation science. Collectively, they emphasize that effective cardiac arrest management depends not only on clinical skill but also on information integrity, digital infrastructure, equitable access, and public awareness.

Table 3 summarizes multidisciplinary and technology-enhanced studies addressing cardiac arrest management through artificial intelligence, telemedicine, data science, and educational innovation. Unlike studies confined to specific stages of the Chain of Survival, these works explore holistic frameworks that integrate prevention, real-time intervention, post-resuscitation care, and societal awareness.

## 5. Education and Training in AI-Enhanced Resuscitation

Education represents a critical pillar in the optimization of cardiac arrest management, bridging the gap between theoretical knowledge, procedural skill, and real-world clinical response. In recent years, technological innovations—particularly in the domains of virtual reality (VR), artificial intelligence (AI), and data-driven simulation—have transformed the landscape of cardiopulmonary resuscitation (CPR) and advanced cardiac life support (ACLS) training. These tools not only enhance the realism and interactivity of educational scenarios but also enable personalized feedback, objective performance assessment, and automated skill tracking across diverse learner populations.

A recent study evaluated the impact of AI-supported voice command interfaces compared to traditional VR controllers in virtual reality–based advanced cardiac life support (ACLS) training [66]. Conducted among anesthesiology students, the research assessed user performance, confidence, and sense of presence during simulation. Both groups improved significantly after training, confirming the educational value of VR simulations. However, participants using AI voice commands achieved lower exam scores despite reporting comparable confidence and engagement levels. Interestingly, an overconfidence bias was observed in the voice command group, suggesting that intuitive interfaces may enhance user immersion without necessarily improving learning outcomes. These findings highlight that while AI-enhanced VR tools offer promising opportunities for medical training, careful optimization of interaction design, feedback systems, and cognitive workload balance is essential to ensure effective skill acquisition in resuscitation education.

Complementary to immersive training, data-driven educational ecosystems are redefining how performance is captured, analyzed, and reused for continuous learning. Constable et al. [67] proposed the creation of a video database of CPR performance to enable interdisciplinary collaboration and AI-based assessment. Their repository, containing 3D multi-angle recordings from 40 participants, integrates expert ratings, self-reported confidence, and demographic metadata. Leveraging this dataset, the authors developed an Automatic Clinical Assessment Tool for Basic Life Support, which employs pose estimation algorithms and deep learning networks to evaluate CPR performance quality objectively. The system detects movement precision, compression depth, and rhythm consistency, aligning AI-generated feedback with expert scoring. Beyond the technical innovation, the authors highlight ethical and legal considerations in educational data sharing—including consent, privacy, and ownership of biometric movement data—emphasizing that educational AI must be grounded in transparent governance and responsible use.

Together, these studies illustrate that AI-enhanced education in resuscitation is not solely about automation but about augmenting human learning through intelligent feedback, multimodal data integration, and ethical stewardship. Virtual environments and shared performance databases can standardize skill acquisition globally, bridge geographic disparities in training quality, and foster real-time analytics for competency-based evaluation.

## 6. Limitations and Future Perspectives

In reviewing the current landscape of AI- and ML-enhanced resuscitation science, several methodological, operational, and ethical limitations must be acknowledged. To facilitate clarity, we summarize the major limitations identified in the literature together with potential approaches to address them in a dedicated table (Table 4). These limitations include restricted generalizability, limited external validation, heterogeneous outcome definitions, real-time implementation constraints, data-quality issues, and insufficient integration with clinical workflows.

A critical dimension of these limitations relates to the ethical implications of deploying AI/ML systems in resuscitative care. The use of such technologies may challenge established ethical principles. Autonomy may be compromised when algorithmic recommendations override or obscure human decision-making. Beneficence and non-maleficence may be threatened by incorrect predictions, poor calibration, or lack of interpretability. Justice is at risk when training datasets underrepresent certain demographic or clinical subgroups, leading to systematic bias and unequal performance across populations.

To address these concerns, several implementation strategies are necessary. These include transparent reporting of model development and validation, incorporation of explainability mechanisms, continuous model monitoring, and governance frameworks ensuring that clinicians retain final decision-making authority. Equally important is improving population representativeness by expanding datasets to include diverse demographic, geographic, and clinical groups; applying bias detection and mitigation techniques; and conducting stratified performance assessments to identify disparities.

Together, these considerations highlight the need for robust, ethically grounded, and clinically validated AI/ML systems that can safely support decision-making across the resuscitation continuum.

Recent advances also demonstrate the growing use of AI, machine learning, and artificial neural networks to predict key clinical outcomes such as return of spontaneous circulation and survival to hospital discharge. Several large cardiac arrest registries, including multinational initiatives such as the European Registry of Cardiac Arrest, provide the scale and heterogeneity needed to develop and validate robust outcome-prediction models.

Despite the significant progress in integrating artificial intelligence (AI) into cardiac arrest management, current research still faces several limitations. Many of the studies reviewed are retrospective or experimental, with limited external validation and heterogeneity in datasets, algorithms, and clinical endpoints. The lack of standardized AI evaluation frameworks complicates the comparison of models and the translation of results into real-world emergency systems. Moreover, most existing AI models are trained on specific populations or registry data, raising concerns regarding generalizability across regions, socioeconomic contexts, and health system infrastructures.

A further limitation lies in the ethical and legal implications of AI implementation. Data privacy, algorithmic bias, accountability, and the transparency of machine learning decision-making remain unresolved challenges. In emergency care, where decisions must be made within seconds, the trust and interpretability of AI predictions are critical for their adoption by clinicians and dispatchers.

From a technical perspective, integrating multimodal data—such as physiological signals, voice analysis, imaging, and geolocation—still requires robust interoperability standards and computational efficiency for real-time deployment. Moreover, while many AI tools demonstrate high accuracy in controlled settings, prospective trials in dynamic prehospital environments are scarce.

Future directions should include large-scale, multicentric validation studies, the development of explainable AI systems that enhance clinical trust, and the creation of international registries for AI-assisted resuscitation research. Emphasis must also be placed on AI ethics education for healthcare professionals and the inclusion of human-centered design principles to ensure equitable, safe, and transparent implementation. Ultimately, the synergy between human expertise and machine intelligence could redefine the entire continuum of cardiac arrest management—from prevention to post-resuscitation recovery.

An additional limitation of the current evidence base is the likelihood of publication bias within the AI/ML literature, as studies reporting positive or high-performing models are more frequently published compared with those reporting negative, neutral, or inconclusive results. This bias may lead to an overestimation of the apparent effectiveness of AI-driven approaches in resuscitation. Future research should therefore prioritize prospective and real-world validation studies, transparent reporting of unsuccessful models, and systematic evaluation frameworks capable of confirming—or disproving—the clinical utility of AI systems across diverse populations and care settings.

## 7. Conclusions

Artificial intelligence and machine learning are increasingly applied across the full resuscitation continuum. Using the six links of the Chain of Survival as the organizing framework, this review highlights the main domains in which AI/ML shows the greatest potential: early recognition and activation, support for high-quality CPR, optimization of advanced resuscitation interventions, and enhanced post-cardiac arrest prognostication. Integrative systems that span multiple stages of care, as well as AI-driven approaches to education and training, represent additional areas of rapid development. Across the entire Chain of Survival, effective education and training serve as a unifying component that supports the performance of every link, reinforcing the essential role of preparedness for both lay responders and clinical teams.

Despite these promising directions, important challenges remain, including issues related to data representativeness, transparency, real-time implementation, and ethical considerations. Continued methodological refinement and rigorous clinical evaluation are essential to ensure that AI/ML systems can be safely and effectively incorporated into resuscitation practice, ultimately contributing to improved patient outcomes.

## Figures and Tables

**Table 1:** Prevention and Preparedness (AI-based studies).

Reference	Objective	Methodology	Key Findings	Relevance to Prevention and Preparedness
Kim et al., 2025 [22]	To evaluate changes in clinical outcomes following implementation of VitalCare (an AI-based early warning system) and validate algorithmic performance	Retrospective analysis of 30,785 inpatient cases using electronic health record data from general wards and ICUs	The AI-based model showed strong predictive capacity for major in-hospital adverse events by providing early warnings to clinicians	Demonstrates how AI early-warning systems enhance preparedness, optimize human resource use, and improve workflow efficiency in hospital settings
Oberdier et al., 2025 [23]	To predict sudden cardiac arrest (SCA) using short 12-lead ECG segments and deep convolutional neural networks	Analysis of public ECG datasets (10 s recordings) from 221 SCA and 1046 control subjects, including demographic and temporal variables	Deep learning achieved promising accuracy in SCA prediction; performance improved as ECGs were recorded closer to the event. QRS complex data were the most influential features	Highlights the feasibility of AI-based ECG analysis for pre-event risk detection and its potential to improve individualized preventive screening
Ghasad et al., 2025 [24]	Systematic review of automated sudden cardiac death (SCD) prediction models developed between 2011–2023	Analysis of ML/DL algorithms (KNN, SVM, decision trees, random forest, CNNs) using databases such as MIT-BIH SCD Holter	Machine and deep learning methods achieved up to 97% accuracy, predicting SCD 30–70 min before onset. However, performance depends on training data size and multimodal integration	Confirms that advanced AI models can anticipate SCD with high precision but emphasizes the need for larger datasets and real-time clinical implementation
Dores et al., 2024 [25]	To review methodologies and challenges in preparticipation screening of athletes to prevent SCD	Narrative review integrating literature on digital screening, ECG interpretation, and AI-enhanced cardiology	AI and digital tools could improve early detection of cardiac abnormalities in athletes, addressing current gaps in manual screening	Underlines the need for ethical and regulatory frameworks to integrate AI safely into preventive cardiology and sports medicine
Ghebran et al., 2022 [15]	To develop an interpretable AI model predicting postoperative ICU requirement in emergency surgery	Optimal classification tree (OCT) algorithm trained on 464,861 cases from the ACS NSQIP database (2007–2017)	The Predictive OpTimal Trees in Emergency Surgery Risk ICU tool accurately predicted postoperative critical care need (C-statistic ≈ 0.89)	Demonstrates AI’s ability to improve perioperative preparedness, ICU triage, and early postoperative intervention to reduce preventable mortality
Chen et al., 2022 [17]	To implement real-time AI-assisted detection of STEMI in prehospital 12-lead ECGs	CNN-LSTM model used by EMTs in 14 AI-equipped fire stations; 362 ECGs analyzed, compared to physician interpretation	AI achieved 99% accuracy (AUC 0.997) and reduced ECG-to-feedback time from 113 s to 37 s, enabling earlier reperfusion	Proves that AI can enhance prehospital preparedness and minimize delays in STEMI treatment through rapid and autonomous triage
El Hechi et al., 2022 [16]	To validate the Trauma Outcomes Predictor model for geriatric trauma patients using ML-based OCTs	Retrospective cohort of 260,505 patients (aged ≥ 65) from the ACS-TQIP database; model evaluated for mortality and morbidity prediction	Model achieved excellent discrimination (C = 0.83–0.92) and accurately predicted mortality and complications	Reinforces AI’s role in early identification of high-risk trauma patients, enhancing system-level readiness and tailored resource deployment
Levandowski et al., 2021 [18]	To develop a rapid response AI-based rescue system (PROTECTOR) for out-of-hospital cardiac arrest detection	Fuzzy logic–based ECG rhythm classifier linked to automatic alarm and geolocation transmission to EMS	The system achieved 100% sensitivity and 97.8% specificity, transmitting alarms and AED maps within seconds	Represents a breakthrough in community-level preparedness, providing immediate detection and automated EMS activation for SCA events
Thorn et al., 2019 [21]	To identify and assess predictive models for acute traumatic coagulopathy (ATC)	Systematic review of studies (1998–2018) evaluating prognostic tools using clinical and laboratory variables	Bayesian network models predicted abnormal coagulation (PT > 1.2) with 90% sensitivity and 92% specificit	Demonstrates AI’s potential in trauma risk prediction and prevention, though external validation is still limited
Olive et al., 2018 [20]	To discuss challenges in monitoring pediatric cardiac ICU patients and AI’s potential for early decompensation prediction	Narrative review of monitoring data and predictive modeling in PCICU environments	AI and big data analytics can anticipate deterioration by integrating physiological, laboratory, and clinical variables	Highlights AI’s preventive role in pediatric intensive care by enhancing surveillance and anticipating decompensation before critical events

**Table 2:** AI-Based Technologies Supporting Emergency Medical System Activation.

Reference	Objective	Methodology	Key Findings	Relevance to EMS Activation
Scquizzato et al., 2021 [26]	To review how emerging technologies are being implemented across all steps of the Chain of Survival and their effects on cardiac arrest outcomes	Narrative review summarizing current and future applications of technology (mobile responders, drones, AI dispatchers, wearables) in out-of-hospital and in-hospital cardiac arrest management	Technology now enhances every stage of the Chain of Survival—from prevention and recognition to CPR and defibrillation. Citizen-alert apps, drones delivering AEDs, and AI-based dispatch support improved bystander response and outcomes	Demonstrates that AI and digital tools shorten EMS activation time, improve early recognition, and optimize system coordination across prehospital and in-hospital settings
Chin et al., 2021 [29]	To develop an AI model capable of assessing callers’ emotional states during out-of-hospital cardiac arrest (OHCA) emergency calls	Analysis of 337 OHCA audio recordings using Mel-frequency cepstral coefficients and support vector machines (SVM). Performance validated by repeated random sub-sampling cross-validation (RRS-CV)	The AI model classified emotional and cooperative levels with >90% predictive accuracy and 98.6% specificity, even when using only the first 10 s of voice input	Facilitates dispatcher prioritization by identifying emotionally stable callers, allowing focus on high-stress interactions where delayed CPR guidance is more likely
Zicari et al., 2021 [30]	To apply the EU “Trustworthy AI” framework (HLEG) to evaluate an ML-based system for recognizing cardiac arrest during emergency calls	Interdisciplinary 1Z-Inspection^®^ involving ethicists, policymakers, technical, and clinical experts; case study based on Copenhagen’s ML-assisted dispatcher system	Highlighted the necessity of human oversight, transparency, and interdisciplinary ethical evaluation in clinical AI deployment	Reinforces that ethically governed AI systems increase trust and reliability in EMS operations, supporting sustainable integration of AI into dispatch systems
Byrsell et al., 2021 [27]	To assess whether ML could improve dispatcher recognition of OHCA within the first minute of emergency calls	Observational study analyzing 851 OHCA calls with ML model tuned for multiple false positive rate (FPR) thresholds	ML recognized 36% of OHCAs within the first minute vs. 25% by dispatchers, reducing median recognition time by 28 s (*p* < 0.001)	Confirms AI’s ability to accelerate OHCA recognition and initiate EMS activation faster, directly enhancing survival chain efficiency
Blomberg et al., 2019 [31]	To compare ML performance with human dispatchers in identifying OHCA from recorded emergency calls	Retrospective analysis of 108,607 emergency calls in Copenhagen; ML trained on audio features and validated against dispatcher decisions	ML achieved higher sensitivity (84.1% vs. 72.5%) and shorter recognition times (median 44 s vs. 54 s)	Establishes ML as a decision-support tool that accelerates dispatcher recognition and shortens EMS activation time
Blomberg et al., 2021 [28]	To evaluate ML-assisted dispatcher alerts in real emergency settings and their impact on OHCA recognition	Double-masked, randomized clinical trial including 169,049 emergency calls (Copenhagen, Denmark). ML alerts compared with standard dispatcher protocols	Dispatchers assisted by ML recognized 93.1% of confirmed OHCA vs. 90.5% in controls (*p* = 0.15). ML alone demonstrated higher sensitivity (85% vs. 77.5%) and faster recognition	Validates the real-world feasibility of AI-assisted dispatch; AI complements human performance by increasing recognition speed and reducing missed cardiac arrests

**Table 3:** Integrative and AI-Driven Perspectives in Resuscitation Science: Cross-Domain Studies Beyond the Conventional Chain of Survival.

Reference	Objective	Methodology	Key Findings	Relevance
Goyal et al., 2025 [48]	To evaluate telemedicine integration in cardiac emergency care	Review	Integrating artificial intelligence enhances telemedicine’s potential by enabling personalized care and predictive analytics	Telemedicine strengthens the “Chain of Survival” for out-of-hospital cardiac arrest through telecommunicator-assisted CPR, boosting bystander CPR rates and survival chances
Islam et al., 2025 [6]	To explore the transformative role of ML and AI in cardiopulmonary resuscitation (CPR)	Systematic survey and taxonomy of ML techniques in CPR	ML techniques classified into four CPR-related domains: rhythm analysis, outcome prediction, compression modeling, and ROSC detection; highlighted XAI for model transparency.	Bridges the gap between resuscitation science and advanced ML, providing a structured foundation for innovation in ML-enhanced CPR
Fijacko et al., 2025 [49]	To analyze topics and technologies represented at resuscitation conferences	Bibliometric analysis using chain-of-survival framework	“Recognition and prevention” dominated conference abstracts; ML used in 54 (Resuscitation 2024) and 47 (Symposium 2024) studies, but no deep learning applied	Identifies research gaps, showing the underrepresentation of advanced AI methods in current resuscitation science
Wei et al., 2025 [50]	To assess ML’s predictive value for cardiac arrest occurrence and outcomes	Systematic review and meta-analysis	ML is a promising tool for predicting cardiac arrest and outcomes such as ROSC and mortality	Supports AI-driven enhancement of traditional prognostic tools for outcome prediction
Lapostolle et al., 2025 [51]	To review technological innovations for cardiac arrest management	Perspective review.	Highlighted emerging tools like AI-assisted dispatch, drone AED delivery, and mobile citizen responder apps	Emphasizes prioritizing early links in the Chain of Survival through gamification and education
Fortunov R.M. et al., 2024 [52]	To examine the integration of AI and automation in perinatal and resuscitation care	Narrative review	Identified key roles for predictive algorithms and remote monitoring in critical care	Suggests AI as an aid for rapid response and workflow optimization in emergent perinatal scenarios
Rietchel et al., 2025 [53]	To discuss point-of-care ultrasound (POCUS) in trauma anesthesiology	Narrative review	Expanding evidence for AI-assisted interpretation of ultrasound during CPR; POCUS use growing globally	Promotes ultrasound accessibility and skill dissemination in low-resource resuscitation environments
Shao et al., 2024 [54]	To introduce an intelligent system for diagnosing and treating IHCA	Hybrid model with deep reinforcement learning and virtual data generation	Improved CPR and ROSC outcomes even with incomplete patient data.	Demonstrates AI’s reliability in managing in-hospital cardiac arrest under uncertainty
Nadasi et al., 2024 [55]	To analyze how Chicago Med portrays medical AI and influences public perception	Qualitative media content analysis.	Presented both benefits and ethical dilemmas of AI in healthcare, from automation bias to equity issues	Highlights media’s role in shaping public trust and critical thinking about medical AI
Semeraro et al., 2024 [56]	To predict future CA management innovations using AI tools	Exploratory literature review with ChatGPT-4 and Gemini Advanced	Predicted adoption of robot CPR, wearable AEDs, and brain–computer interfaces within 3–8 years	Calls for ethical oversight and interdisciplinary collaboration in implementing AI-driven innovations
Bikun et al., 2024 [57]	To map misinformation about CPR and first aid	PRISMA-based scoping review	97.7% of public sources contained misinformation; 25% included harmful or misleading advice	Urges global strategies to detect and prevent misinformation in public CPR education
Plodr et al., 2024 [58]	To review current trends in OHCA management	Narrative review	Emphasized improving EMDC information flow and rapid recognition of life-threatening conditions	Reinforces communication efficiency as a determinant of prehospital survival
Aquel et al., 2023 [59]	To examine AI and ML in predicting and managing sudden cardiac arrest outcomes	Literature synthesis	AI enhances prediction of shockable rhythms and neurological outcomes; supports real-time feedback.	Collaboration among clinicians, data scientists, and regulators is essential for optimizing AI-based CPR
Piliuk et al., 2023 [60]	To review AI applications and challenges in emergency medicine	Systematic review of 380 studies (116 included)	Found isolated, small-scale AI studies with limited generalization	Advocates for unified, human-AI integrated systems across emergency care
Carrigan et al., 2023 [61]	To assess the digital hospital model in cardiac and pulmonary care	Scoping review (13 studies)	Wireless ECG telemonitoring improved 24 h survival but not discharge rates	Demonstrates potential benefits of digital infrastructure, requiring robust support systems
Moon et al., 2023 [62]	To analyze spatial correlations in OHCA outcomes using ML	Retrospective registry analysis using VAE and DPMM clustering	Identified 8 clusters by geography; transfer to higher-level centers predicted better survival	Provides a data-driven framework for regional optimization of emergency systems
Batey et al., 2024 [63]	To apply AI and computer vision in neonatal resuscitation research	Neural network training on neonatal resuscitation videos	Real-time pattern recognition could assist clinicians and improve newborn outcomes	AI-based video feedback may enhance both clinical performance and staff training
Rajagopalan et al., 2022 [64]	To review SCA prevention and awareness initiatives	Narrative review	Highlighted campaigns for CPR education and AED access; discussed AI and voice-command CPR aids	Supports technology-enhanced public training and equitable access to life-saving interventions
Narayan et al., 2019 [65]	To outline global challenges in sudden cardiac arrest management	Review	Proposed a “Respond–Understand–Predict–Prevent” model integrating AI and open data	Advocates a holistic, policy-oriented approach to SCA prevention and research

**Table 4:** Major Limitations of AI/ML Systems in Resuscitation and Proposed Mitigation Strategies.

Limitation	Description/Risks	Suggested Approaches to Address the Limitation
**1. Limited generalizability and external validation:** Perform external and temporal validation; Conduct prospective and real-world studies.	Many models are trained on small or single-center datasets, reducing applicability across settings	Use multicenter, multinational datasets
**2. Underrepresentation of key demographic or clinical subgroups (bias)** Report model performance across subgroups; Apply fairness metrics and bias-correction techniques; Collaborate internationally to improve inclusiveness.	Certain populations (e.g., elderly, women, minorities, trauma, drowning, pediatrics) are often insufficiently represented, leading to biased predictions	Increase dataset diversity and stratified sampling
**3. Limited interpretability (“black-box” behavior)** Provide clinical rationale aligned with guidelines; Develop interpretable-by-design architectures.	Lack of transparency reduces clinician trust and may hinder adoption; may impair ethical autonomy and informed decision-making	Incorporate explainable AI (XAI) tools
**4. Risk of violating ethical principles (autonomy, beneficence, non-maleficence, justice)** Continuous performance monitoring; Ethical review committees for model deployment; Standardized governance frameworks.	Algorithmic recommendations may override clinician judgment (autonomy); inaccurate models may harm patients (maleficence); biased models create inequity (justice)	Maintain clinician-in-the-loop decision-making
**5. Heterogeneous definitions of outcomes (survival, neurologic recovery)** Report primary and secondary outcomes clearly; Encourage consensus-based outcome reporting.	Lack of uniform outcome metrics complicates model comparison and reproducibility	Adhere to standardized definitions
**6. Data quality limitations (missing data, noise, lack of labels)** Use active learning and semi-supervised approaches; Improve data collection infrastructure.	Inaccurate or incomplete data degrade model performance and reliability	Implement robust preprocessing and imputation pipelines
**7. Limited real-time feasibility and integration with clinical workflows** Test models in simulated and real-time environments; Co-design solutions with EMS and clinical teams.	Models may not function adequately during real-time CPR; integration into EMS and hospital systems remains problematic.	Optimize computational efficiency
**8. Human–AI interaction challenges** User-centered interface design; Continuous feedback loops to align AI behavior with user needs.	Users may misunderstand or misuse AI outputs, affecting safety; risk of over-reliance or under-reliance	Training programs for clinicians and lay responders
**9. Regulatory uncertainty and lack of standardized approval pathways** Post-market surveillance; Transparent documentation and model auditing.	Inconsistent regulation across regions impedes safe deployment.	Develop clear regulatory pathways (FDA/EMA)
**10. Limited evidence on long-term outcomes and impact on survival** Collect long-term survival and neurologic outcomes; Evaluate system-level impacts.	Most studies evaluate accuracy metrics, not patient-centered outcomes.	Conduct clinical trials and implementation studies

## Data Availability

Not applicable.
